# Insulin-like growth factor axis in pregnancies affected by fetal growth disorders

**DOI:** 10.1186/s13148-016-0178-5

**Published:** 2016-01-27

**Authors:** Aamod R. Nawathe, Mark Christian, Sung Hye Kim, Mark Johnson, Makrina D. Savvidou, Vasso Terzidou

**Affiliations:** Imperial College London, London, UK; Academic Department of Obstetrics and Gynaecology, Chelsea and Westminster Hospital, London, UK; Warwick Medical School, University of Warwick, Coventry, UK

**Keywords:** Insulin growth factor, Small fetus, Large fetus, Placental expression, DNA methylation

## Abstract

**Background:**

Insulin-like growth factors 1 and 2 (IGF1 and IGF2) and their binding proteins (IGFBPs) are expressed in the placenta and known to regulate fetal growth. DNA methylation is an epigenetic mechanism which involves addition of methyl group to a cytosine base in the DNA forming a methylated cytosine-phosphate-guanine (CpG) dinucleotide which is known to silence gene expression. This silences gene expression, potentially altering the expression of IGFs and their binding proteins. This study investigates the relationship between DNA methylation of components of the IGF axis in the placenta and disorders in fetal growth. Placental samples were obtained from cord insertions immediately after delivery from appropriate, small (defined as birthweight <10th percentile for the gestation [SGA]) and macrosomic (defined as birthweight > the 90th percentile for the gestation [LGA]) neonates. Placental DNA methylation, mRNA expression and protein levels of components of the IGF axis were determined by pyrosequencing, rtPCR and Western blotting.

**Results:**

In the placenta from small for gestational age (SGA) neonates (*n* = 16), mRNA and protein levels of IGF1 were lower and of IGFBPs (1, 2, 3, 4 and 7) were higher (*p* < 0.05) compared to appropriately grown neonates (*n* = 37). In contrast, in the placenta from large for gestational age (LGA) neonates (*n* = 20), mRNA and protein levels of IGF1 was not different and those of IGFBPs (1, 2, 3 and 4) were lower (*p* < 0.05) compared to appropriately grown neonates. Compared to appropriately grown neonates, CpG methylation of the promoter regions of IGF1 was higher in SGA neonates. The CpG methylation of the promoter regions of IGFBP1, IGFBP2, IGFBP3, IGFBP4 and IGFBP7 was lower in the placenta from SGA neonates as compared to appropriately grown neonates, but was unchanged in the placenta from LGA neonates.

**Conclusions:**

Our results suggest that changes in CpG methylation contribute to the changes in gene expression of components of the IGF axis in fetal growth disorders. Differential methylation of the IGF1 gene and its binding proteins is likely to play a role in the pathogenesis of SGA neonates.

## Background

Insulin-like growth factors 1 and 2 (IGF1, IGF2) are expressed in the placenta and are known to regulate fetal growth [1]. While maternal IGF1 has been shown to stimulate fetal growth by increasing the transfer of nutrients to the fetus [2], fetal IGF1 is presumed to stimulate fetal growth by promoting anabolic events and DNA synthesis [3]. IGF1 gene ablation has been shown to reduce fetal weight while IGF1 administration has been shown to increase fetal weight [4]. The main role of IGF2 appears to be mediated through its effects on cellular growth and tissue-specific cell proliferation [5]. IGF2 overexpression in mice causes placental and fetal overgrowth, whereas IGF2 gene deletion reduces placental and fetal weight [6, 7]. When both genes are deleted simultaneously, the effects on fetal growth are additive [8]. Studies on placental IGF1 and IGF2 expression in growth-restricted fetuses are inconsistent [9–16]. The bioavailability of IGFs is modulated by their seven binding proteins (IGFBP) [17]. The affinity of IGFBP3 for IGFs is higher than other IGFBPs [18], hence approximately 80–90 % of IGFs are bound to IGFBP3 [19]. In pregnancy, the IGFBPs and in particular IGFBP2, 3, 4 and 5, but not IGFBP1, are cleaved by proteases, which reduce their affinity for IGFs [20, 21]. As a result, the levels of maternal IGFBP1 continue to increase during pregnancy in a similar fashion to those of IGF1 [22]. Although IGFBP3 is the most common binding protein found in the placenta [23], IGF1 and IGFBP1 appear to play the major role in regulating fetal growth. IGFBP1 affinity towards IGF1 is three times higher than proteolyzed IGFBP3, but is lower for IGF2 [19]. Hence, the increased activity of proteases during pregnancy shifts the control of IGF action from IGFBP3 to IGFBP1.

Placental methylation is significantly lower compared to other somatic tissues [24–26] and this has been associated with promoting fetal development throughout gestation [27–30]. Placental function and the intrauterine environment play critical roles in fetal programming [31–33], and different lines of evidence suggest a role for epigenetic mechanisms, including genomic imprinting and DNA methylation in this process [34, 35]. Several animal models have also suggested that altering placental DNA methylation plays an important role in placental and fetal growth [36].

The aim of the current study was to investigate the genetic and epigenetic changes in placental IGF1/IGF2 and their seven binding proteins in order to understand the “net IGF bioavailability” in pregnancies with small for gestational age (SGA), large for gestational age (LGA) and appropriately grown neonates.

## Results

### Maternal and neonatal characteristics

Seventy-four women were recruited to the study, with appropriately grown (*n* = 38), SGA (*n* = 16) and LGA (*n* = 20) neonates. The placental mRNA and protein expression was analysed in all cases. DNA methylation levels were analysed in a total of 24 women (8 in each group). The maternal and pregnancy characteristics of the study participants are given in Table 1. Compared to appropriately grown neonates, women with SGA neonates were delivered earlier, and women with LGA neonates had a higher BMI (Table 1).

**Table 1:** Maternal and pregnancy characteristics

Variables	Appropriately grown (*n* = 38)	Small for gestational age (*n* = 16)	Large for gestational age (*n* = 20)	*p* value (overall)
Maternal age (years)	31.5 (28.7–35.0)	31.5 (25.2–33.7)	33.0 (30.0–37.2)	0.173
Parity, *n* (%)				
Nulliparous, *n* (%)	20 (52.6)	10 (62.5)	11 (55.0)	0.8
Parous, *n* (%)	18 (47.4)	6 (37.5)	9 (45.0)	
Racial origin				
Caucasian, *n* (%)	25 (65.8)	11 (68.8)	19 (95.0)	0.045
Black, *n* (%)	7 (18.4)	3 (18.8)	1 (5.0)	
Other, (%)	6 (15.8)	2 (12.4)	0 (0)	
Smokers, *n* (%)	1 (2.6)	2 (12.5)	1 (5)	0.341
Maternal body mass index at booking	23.0 (20.0–25.2)	22.0 (20.2–24.0)	26.5 (23.2–36.0)*	0.002
Gestational age at delivery (weeks)	39.2 (39.0–41.0)	36.5 (32.2–38.8)*	39.5 (39.0–40.0)	0.001
Maternal age (years)	31.5 (28.7–35.0)	31.5 (25.2–33.7)	33.0 (30.0–37.2)	0.173
Parity, *n* (%)				
Nulliparous, *n* (%)	20 (52.6)	10 (62.5)	11 (55.0)	0.8
Parous, *n* (%)	18 (47.4)	6 (37.5)	9 (45.0)	
Birthweight percentile	54.5 (21.0–76.2)	1.4 (0.3–6.8)*	98.3 (95.2–99.6)*	<0.001

Data are expressed as median (interquartile range). Comparisons between categorical and continuous variables were done by *x* ^2^ or Fisher’s exact test and Mann-Whitney test both with post hoc Bonferroni correction. *p < 0.01 for comparisons vs. appropriately grown neonates

### Placental mRNA expression and protein expression of the IGF axis and its binding proteins

**Fig. 1:**
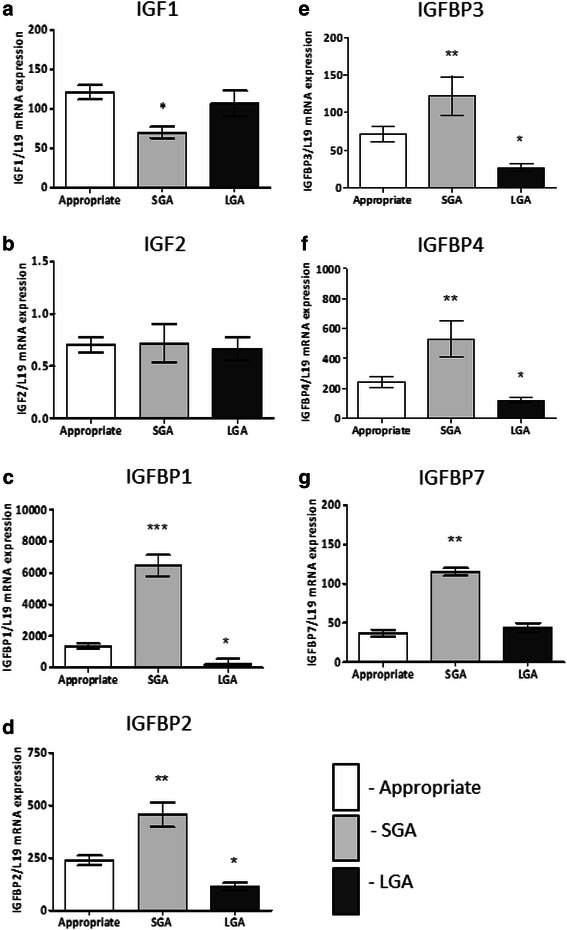
Placental mRNA expression of the IGF axis in appropriately grown, SGA and LGA neonates. **a** IGF1 expression, **b** IGF2 expression, **c** IGFBP1 expression, **d** IGFBP2 expression, **e** IGFBP3 expression, **f** IGFBP4 expression, **g** IGFBP7 expression. *p < 0.05, **p < 0.005, ***p < 0.0005 when compared to appropriately grown neonates; *SGA* small for gestational age, *LGA* large for gestational age

**Fig. 2:**
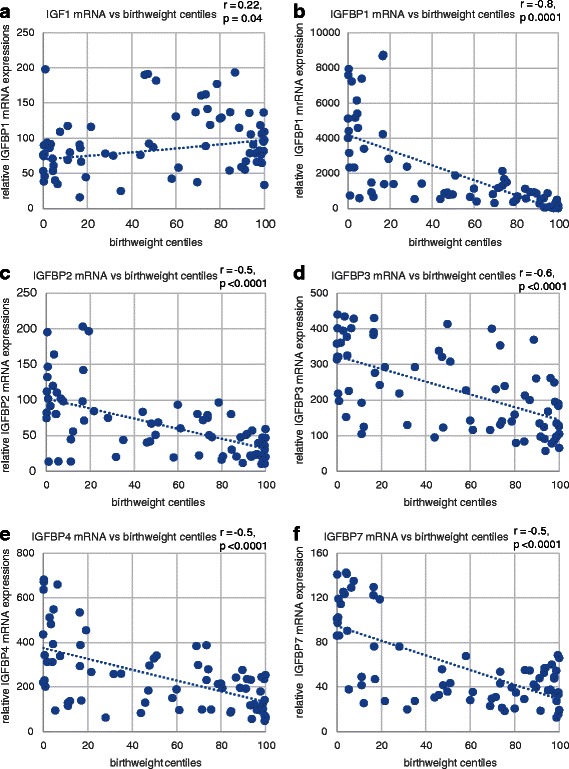
Spearman’s correlation between placental mRNA expression and increasing order of birthweight centiles. **a** IGF1 expression vs. birthweight centiles, **b** IGFBP1 expression vs. birthweight centiles, **c** IGFBP2 expression vs. birthweight centiles, **d** IGFBP3 expression vs. birthweight centiles, **e** IGFBP4 expression vs. birthweight centiles, **f** IGFBP7 expression vs. birthweight centiles. A significant positive correlation between IGF1 mRNA expression and a significant negative correlation between the binding protein expression with increasing order of birthweight centiles suggest the crucial role played by their inverse relationship in controlling fetal growth

Compared to appropriately grown neonates, the placental mRNA expression of IGF1 was reduced in the SGA group but not in the LGA group (Fig. 1). There were no significant differences in IGF2 gene expression between the groups. Compared to appropriately grown neonates, the IGFBP1, 2, 3, and 4 gene expression was significantly higher in the SGA group (*p <* 0.001) and lower in the LGA group (*p <* 0.05). IGFBP5 and IGFBP6 expression was not detected in the placenta samples. IGFBP7 expression was higher in SGA group (*p <* 0.001) and not different in the LGA group, compared to appropriately grown neonates. The mRNA expression of IGF1 was positively correlated with birthweight centiles (*r =* 0.22, *p =* 0.04) (Fig. 2). Conversely, there was a significant negative correlation between all the binding protein gene expression and birthweight percentiles (Fig. 2).

**Fig. 3:**
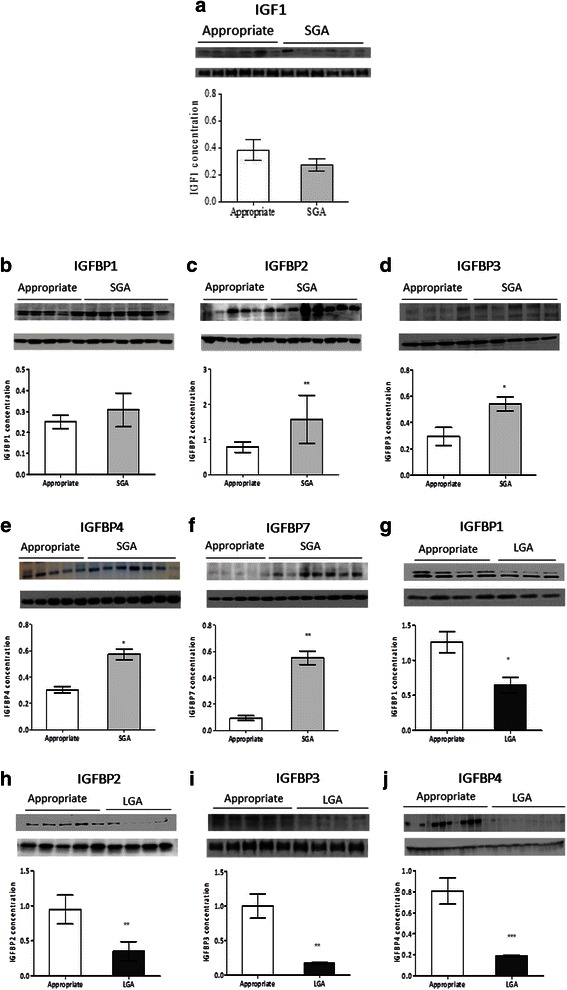
Placental protein expression of the IGF axis in appropriately grown, SGA and LGA neonates. **a** IGF1 expression, **b** IGFBP1 expression, **c** IGFBP2 expression, **d** IGFBP3 expression, **e** IGFBP4 expression, **f** IGFBP7 expression, **g** IGFBP1 expression, **h** IGFBP2 expression, **i** IGFBP3 expression, **j** IGFBP4 expression. *p < 0.05, **p < 0.005, ***p < 0.0005, when compared to appropriately grown neonates. *SGA* small for gestational age, *LGA* large for gestational age. Increased protein expression of IGFBP1, IGFBP2, IGFBP3, IGFBP4 and IGFBP7 in SGAs and decreased expression of IGFBP1, IGFBP2, IGFBP3 and IGFBP4 in LGA neonates suggest inverse relationship of the binding protein expression with fetal growth

Western blotting revealed a correlation between placental protein expression and changes seen in the mRNA expression. Compared to appropriately grown neonates, there was a tendency for a lower placental IGF1 protein in SGA group (Fig. 3a). The protein content of IGFBP2, IGFBP3, IGFBP4 and IGFBP7 genes was significantly higher in SGA group (IGFBP2, *p* < 0.005; IGFBP3, *p* < 0.05; IGFBP4, *p* < 0.05; IGFBP7, *p* < 0.05) while that of IGFBP1, IGFBP2, IGFBP3 and IGFBP4 was lower in the LGA group (IGFBP1, *p* < 0.05; IGFBP2, *p* < 0.005; IGFBP3, *p* < 0.005; IGFBP4*, p* < 0.0005) (Fig. 3), compared to appropriately grown neonates.

### Promoter CpG methylation of the IGF axis and its binding proteins

**Fig. 4:**
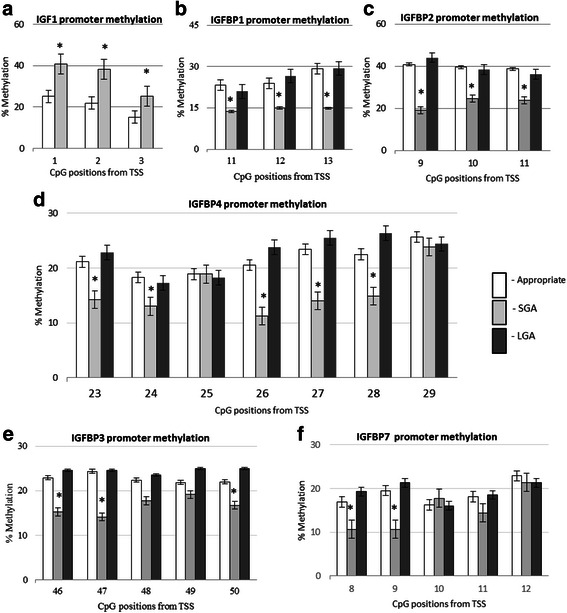
Placental promoter methylation in appropriately grown, SGA and LGA neonates. **a** IGFF1 promoter methylation—CpG sites 1, 2 and 3; **b** IGFBP1 promoter methylation—CpG sites 11, 12 and 13, **c** IGFBP2 promoter methylation—CpG sites 9, 10, 11; **d** IGFBP4 promoter methylation—CpG sites 23, 24, 25, 26, 27, 28 and 29; **e** IGFBP3 promoter methylation—CpG sites 46, 47, 48, 49 and 50; **f** IGFBP7 promoter methylation—CpG sites 8, 9, 10, 11 and 12. *p < 0.05 when compared to appropriately grown, *SGA* small for gestational age, *LGA* large for gestational age

**Fig. 5:**
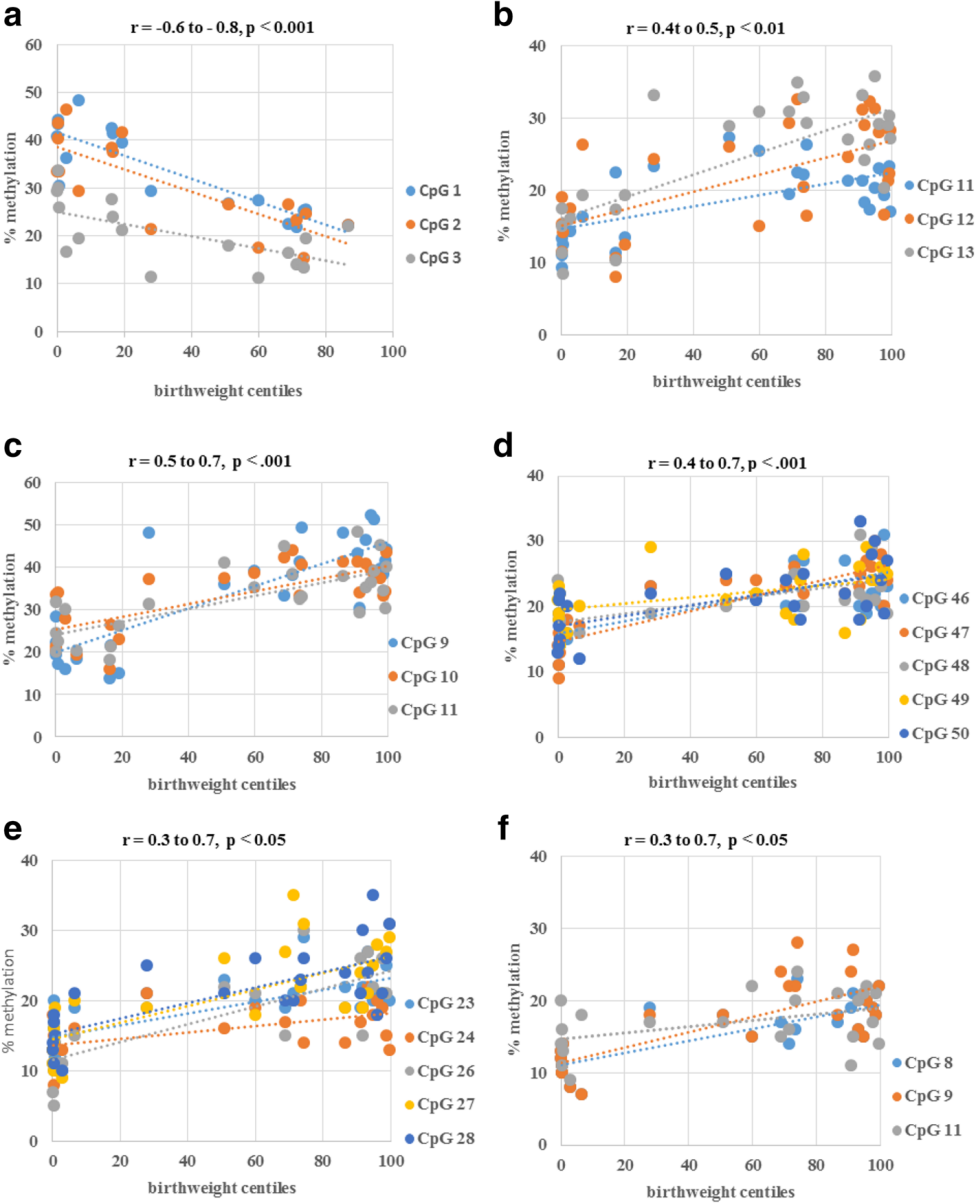
Spearman’s correlation between placental promoter CpG methylation and increasing order of birthweight centiles. **a** IGF1 methylation vs. birthweight centiles, **b** IGFBP1 methylation vs. birthweight centiles, **c** IGFBP2 methylation vs. birthweight centiles, **d** IGFBP3 methylation vs. birthweight centiles, **e** IGFBP4 methylation vs. birthweight centiles, **f** IGFBP7 methylation vs. birthweight centiles. A significant negative correlation between IGF1 mRNA expression and a significant positive correlation between the binding protein expression with increasing order of birthweight centiles could reflect the indirect role played by DNA methylation relationship in controlling fetal growth by controlling gene expression of the IGF axis

In the SGA group, all CpG sites at the IGF1 promoter were found to be about 1.5 times hypermethylated as compared to appropriately grown neonates (Fig. 4). Conversely, the three CpG sites at the IGFBP1 and IGFBP2 promoters were found to be about half as methylated as the appropriately grown neonates, but there were no methylation differences in the LGA group. The CpG sites in the IGFBP3 promoter at positions 46, 47 and 50 were also significantly hypomethylated in the SGA with no differences in the LGA group (Fig. 4). However, there were no methylation differences at sites 48 and 49 in the IGFBP3 promoter across all the groups. CpG sites 23, 24, 26, 27 and 28 but not 25 and 29 in the IGFBP4 promoter and sites 9 and 10 in the IGFBP7 promoter were significantly hypomethylated in the SGA group, compared to appropriately grown neonates, while there were no methylation differences in the LGA group (Fig. 4). The majority of CpG sites on the IGF1 promoter had a significant negative correlation with IGF1 mRNA expression (CpG 1, *r* = −0.6, *p* = 0.007; CpG 2, *r* = −0.73, *p* = 0.0007; CpG 3, *r* = −0.4, *p* = 0.14) (Table 2). In addition, the majority of the CpG sites on the promoters of the binding proteins IGFBP1, IGFBP2 and IGFBP3, CpG sites 23, 26, 27, 28 and 29 on the IGFBP4 and CpG sites 8 and 9 on the IGFBP7 promoter had a significant negative correlation with their respective mRNA expression (Table 2). No significant correlation with their respective mRNA expression was identified between CpG sites 24 and 25 of IGFBP4 promoter and sites 10, 11 and 12 of IGFBP7 promoter. Birthweight centiles had a significant negative correlation with IGF1 promoter methylation but a significant positive correlation with binding protein methylation (Fig. 5).

**Table 2:** Correlation between DNA methylation and mRNA expression

Gene	CpG number	CpG location	Rs	*p* value
IGF1	1	Chr12: −102874567	−0.6275	0.007*
IGF1	2	Chr12: −102874689	−0.7377	0.0007*
IGF1	3	Chr12: −102874755	−0.4044	0.10
IGFBP1	11	Chr7: 45927583	−0.4728	0.01*
IGFBP1	12	Chr7: 45927579	−0.6349	0.0005*
IGFBP1	13	Chr7: 45927575	−0.7005	<0.0001*
IGFBP2	9	Chr2: 217497928	−0.7785	<0.0001*
IGFBP2	10	Chr2: 217497846	−0.5856	0.001*
IGFBP2	11	Chr2: 217497740	−0.6205	0.0007*
IGFBP3	46	Chr7: −45960433	−0.8086	<0.0001*
IGFBP3	47	Chr7: −45960418	−0.6627	0.0002*
IGFBP3	48	Chr7: −45960415	−0.5104	0.007*
IGFBP3	49	Chr7: −45960400	−0.4161	0.04*
IGFBP3	50	Chr7: −45960394	−0.6306	0.001*
IGFBP4	23	Chr17: 38599317	−0.5971	0.002*
IGFBP4	24	Chr17: 38599314	−0.3923	0.05
IGFBP4	25	Chr17: 38599311	0.04471	0.83
IGFBP4	26	Chr17: 38599308	−0.6726	0.0003*
IGFBP4	27	Chr17: 38599305	0.626	0.001*
IGFBP4	28	Chr17: 38599302	−0.5158	0.009*
IGFBP4	29	Chr17: 38599299	−0.1613	0.45*
IGFBP7	8	Chr4: −57976141	−0.6501	0.0006*
IGFBP7	9	Chr4: −57976145	−0.6882	0.0002*
IGFBP7	10	Chr4: −57976152	0.19	0.37
IGFBP7	11	Chr4: −57976157	−0.2764	0.19
IGFBP7	12	Chr4: −57976172	−0.05846	0.78

Spearman’s correlation between mRNA expression and DNA methylation showed significant inverse correlation at all CpGs tested except in two locations (24 and 25) at IGFBP4 promoter region and at three locations (10, 11 and 12) at IGFBP7 promoter region

## Discussion

Our study has demonstrated that in pregnancies affected by SGA, the placental IGF and IGFBP axis is altered; we found that placental IGF1 mRNA is decreased, the IGFBPs expression is increased and these changes are associated with alterations in DNA methylation levels of IGF1 (hypermethylated) and IGFBPs (hypomethylated). These findings indicate that epigenetic modification may play a key role in controlling fetal growth. In contrast, in LGA pregnancies, the differences in gene expression could not be explained by corresponding changes in the methylation of the respective gene promoters.

Various reports have described differences in placental IGF and IGFBP mRNA expression in fetuses affected by growth disorders. However, these studies were limited by the small sample size, the number of binding proteins being investigated and the type of tissue being examined i.e. either the placenta [10–12, 37, 38] or umbilical blood [39–41]. In our study, we have assessed the placental IGF axis at the epigenetic, transcriptional and translation levels in pregnancies resulting in appropriate, small and large neonates. The crucial role played by IGF1 in fetal growth and programming is supported by animal studies where IGF1 knockout mice not only had a reduced birthweight but also continued to weigh less throughout their lives [7]. We found IGF1 mRNA expression was lower in SGA group, which is consistent with previous studies [10, 26]. It is well known that altered DNA methylation can contribute to disease pathogenesis [42, 43]. Indeed, we found that the IGF1 gene promoter was hypermethylated in the placenta of SGA neonates. Importantly, IGF1 mRNA expression had a significant positive correlation with birthweight, while IGF1 promoter methylation had a significant negative correlation. These relationships between gene expression, promoter methylation and birthweight suggest that the epigenetic control of IGF1 expression has a causative role in the pathogenesis of fetal growth restriction. In agreement with other studies, we found that IGF1 was not differentially expressed in LGA neonates and IGF2 expression was similar across all the subgroups [44, 45].

We found elevated mRNA and protein levels of IGFBP1, IGFBP2, IGFBP3, IGFBP4 and IGFBP7 in placenta of SGA neonates and decreased expression of IGFBP1, IGFBP2, IGFBP3 and IGFBP4 in the LGA group. Corresponding to these changes in gene expression, the promoters of the respective genes were hypomethylated in the SGA. There was a significant negative correlation between IGFBPs 1, 2, 3, 4 and 7 mRNA expression and birthweight while promoter methylation of all the binding proteins showed a significant positive correlation with it. While some studies have shown an inverse correlation of IGFBP1 and IGFBP2 with birthweight [38, 46], there are conflicting data regarding IGFBP3 gene expression and a paucity of data describing IGFBP4, IGFBP5, and IGFBP6 expression in the placenta of small or large neonates [39, 47, 48]. IGFBP5 and IGFBP6 could not be detected in our samples; this may be due to their expression being restricted to the decidua [1, 49]. IGFBP7 gene expression has not been previously investigated in small or large neonates and has not been detected in human placenta. An inverse correlation between promoter methylation and mRNA expression suggests that DNA methylation plays a crucial role in the gene expression of the binding proteins and therefore impacting in the pathogenesis of small fetal size. There were no methylation differences in the promoters of IGF1 and the binding proteins in the LGA group. It is possible that DNA methylation plays an important role in the pathogenesis of small fetal size while other mechanisms, like microRNA or histone modifications, may be important in the pathogenesis of macrosomia.

Another modification of cytosine is called 5-hydroxymethylcytosine (5-HMC) which is also known to play a role in activating or silencing genes [50]. Liu et al. have recently shown significant differences in 5-HMC concentrations in different tissues of the human body with the highest concentration (0.4–0.6 %) found in the brain, liver, kidney and colorectal tissues while lowest concentration (0.02 %) found in the placenta [51]. Piyasena et al. [52] have suggested that placental 5-methylcytocine and 5-hydroxythethylcytocine patterns are associated with the expression of imprinted genes linked with birthweight. Future work can investigate placental 5-HMC in the IGF system-related genes in appropriately grown, small and large neonates.

## Conclusions

We have found that IGF1 gene is underexpressed and IGFBP1, IGFBP2, IGFBP3, IGFBP4 and IGFBP7 are overexpressed in the placenta of SGA neonates, and these differences could be partly explained by the inverse changes in promoter methylation. In contrast, placental IGFBP1, IGFBP2, IGFBP3 and IGFBP4 genes are underexpressed in pregnancies with LGA neonates, but these differences are not associated with changes in DNA methylation; further studies are required to identify the mechanisms responsible for their gene expression.

## Methods

We obtained placental biopsies, immediately following delivery, from women that delivered appropriate size neonates, small for gestational age (SGA) neonates, defined as birthweight <10th percentile for gestation, adjusted for gestational age, and large for gestational age (LGA) neonates, defined as birthweight >90th percentile for gestation, adjusted for gestational age [53]. Placental samples were collected from the fetal side and washed with PBS to remove contaminants. Areas which appeared calcified or encored were excluded, and samples were stored at −80 °C until further analysis. Umbilical cord tissue, amniotic membranes and decidua were excluded. All pregnancies were dated by a first trimester scan at 11–13 weeks of gestation. Booking maternal body mass index (BMI) was calculated as weight/height in square meter. All patients gave a written informed consent form, and the study was approved by the NRES Committee London—Central REC ref number: 11/LO/1315.

### RNA extraction, cDNA preparation and real-time PCR

RNA extraction was performed using RNeasy Mini Kit (Qiagen, Germany). Any contaminating DNA was removed by DNaseI (Invitrogen) treatment room temperature for 15 min, and complementary deoxyribonucleic acid (cDNA) was prepared by using the MMLV kit (Sigma®, Cat No. M1302) according to the manufacturer’s instructions by reverse transcription of 1 μg of RNA. The cDNA was stored at −20 °C until further analysis. A 2 μl of cDNA was added to 8 μl of reaction mix to make a total of 10 μl per well. The reaction mix consisted of 5 μl of SYBR Green {Sigma®, Cat No QR0100}, 0.2 μl of ROX Reference Dye (Sigma®, Cat No R4526), 300 nM of each primer, and RNase free water to make up the remaining volume. All primers were optimised prior to cDNA amplification. Four such wells were cleaned up using the QIAquick PCR purification kit according to the manufacturer’s instructions (Qiagen®). Serial dilutions from the purified DNA in the ratio of 1:10 were made which were then used in duplicate in the real-time PCR reactions for the respective gene. The steps of the quantification PCR included initial denaturation at 94 °C for 2 min following which samples were subjected to 40 amplification cycles comprising of denaturation at 95 °C for 15 s, annealing and elongation of each gene for 60 s. All the mRNA data was expressed as a relative quantification to the total amount of similarly expressed L19 gene. The results of the melt curve analysis were verified further by electrophoresis on 1 % agarose gel and photographed on UV light illuminator (Clare Chemical Research). A negative control was included per gene in the reaction.

### Primers

The primers were designed using the protein encoding transcripts from www.ensembl.org [54] and Primer3 (version 0.4.0) at http://frodo.wi.mit.edu/. All the primers were between 80 and 150 bp. The primers were checked using Primer-BLAST at http://blast.ncbi.nlm.nih.gov/ to ensure 100 % maximum identification of the nucleotide sequences. The primers were IGF1F:5′-CAGCAGTCTTCCAACCCAT-3′, IGF1R: 5′-ACAGCGCCAGGTAGAAGAG-3′; IGF2F:5′-CAATATGACACCACCGTGCT-3′, IGF2R:5′-GGACTGCTTCCAGGTGTCAT-3′; IGFBP1F:5′-CTGCCAAACTGCAACAAGAA-3′, IGFBP1R:5′-GAGACCCAGGGATCCTCTTC-3′; IGFBP2F:5′-ATGGCGATGACCATCAGA-3′, IGFBP2R:5′-ACCTGGTCCAGTTCCTGTTG-3′; IGFBP3F:5′-CAGAGACTCGAGCACAGCAC-3′, IGFBP3R:5′-GCCGCCTAAGTCACAAAGTC-3′; IGFBP4F:5′-CCCACGAGGACCTCTACATC-3′, IGFBP4R:5′-ATCCAGAGCTGGGTGACACT-3′; IGFBP5F:5′-AGCAGCAACGTTGAGTGATG-3′, IGFBP5R:5′-GATGAAATGAGTGGCGTCCT-3′; IGFBP6F:5′-GCTGTTGCAGAGGAGAATCC-3′, IGFBP6R:5′-GGTAGAAGCCTCGATGGTCA; IGFBP7F:5′-CATCCAATTCCCAAGGACAG-3′, IGFBP7R:5′-TATAGCTCGGCACCTTCACC-3′; L19F:5′-GCGGAAGGGTACAGCCAAT-3′, L19R:5′-GCAGCCGGCGCAAA-3′.

### Protein extraction, western blot and immunodetection

Placental samples were homogenised in modified RIPA buffer containing 1 % Triton ×100, 1 % sodium deoxycholate, 0.1 % SDS, 150 mM sodium chloride, 10 mM Tris (pH 7.4) and 1 mM EDTA with 1 mM of PMSF and protease inhibitor cocktail (Sigma-Aldrich) which was used to lyse the placental samples. The lysate was centrifuged at 13,000×*g* for 30 min at 4 °C and the supernatant extracted to obtain whole-cell protein. The protein extracts were quantified by the Lowry method (Bio-Rad). Protein samples were aliquoted and then stored at −80 °C to avoid freeze-thaw cycles. Denaturation of proteins was performed at 80 °C for 10 min with loading dye consisting of 3 % glycerol, 3 % SDS, 1 % Bromophenol blue and β-mercaptoethanol. Equal amount of proteins (50 μg) were run either on a 10 % SDS-Polyacrylamide gel for 80 min at 120 V, and were transferred to polyvinylidene difluoride (PVDF) membranes (Millipore) for 90 min at 300 mA or on a Bio-Rad Criterion TGX gel, 26-well midi gel (Cat No. 567-1085) for 80 min at 120 V and transferred for 7 min on a Tran-Blot® Turbo™ Midi PVDF Transfer Pack (Cat No. 170-4157). Membranes were incubated in 5 % blocking buffer for 1 h at room temperature and hybridized with primary antibody overnight at 4 °C. Secondary antibody incubation was then carried out the following day and immunodetection using ECL2 or ECL (Fisher Scientific). The primary antibodies were IGF1—Abcam, cat no. Ab9572; IGFBP1—Santa Cruz, cat no. Sc-55474, IGFBP2—Abcam, cat no. Ab109284; IGFBP3—Santa Cruz, cat no. Sc-6004; IGFBP4—Santa Cruz, cat no. Sc-6005; IGFBP7—Santa Cruz, cat no. Sc-13095; β-actin—Abcam, cat no. Ab6276; positive control—liver (human) tissue lysate—Abcam, cat no. Ab29889.

### Gene promoter assays

The CpG assays were designed using the Pyromark CpG software (Qiagen), and the genomic sequences were extracted from the USCS genome browser at www.genome.uscs.edu [55]. The CpGs were identified from the promoter region of each of the target gene. The promoter regions were identified from sequences up to 500 bp upstream of the transcription start sites (TSS) (Table 3). To address epigenetic events associated with fetal growth disorders, we analysed CpG methylation at three sites in the IGF1, IGFBP1 and IGFBP2 promoter regions, five sites in IGFBP3 and IGFBP7 and seven sites in the IGFBP4 promoter regions (Table 2).

**Table 3:** Localisation of the CpG sites in the genome

Gene	CpG number	CpG location	Transcription start site (TSS)	Forward primer	Reverse primer (biotinylated)
IGF1	1	Chr12: −102874567	102874323	GATAGGAAATAGTTGGGGGAATATTTGT	AATCTACTTTACCCCAATCACTTCAA
IGF1	2	Chr12: −102874689	102874323	GATAGGAAATAGTTGGGGGAATATTTGT	AATCTACTTTACCCCAATCACTTCAA
IGF1	3	Chr12: −102874755	102874323	GATAGGAAATAGTTGGGGGAATATTTGT	AATCTACTTTACCCCAATCACTTCAA
IGFBP1	1	Chr7: 45927583	45928093	TTGAGTAGGGTTTTGGGTGTATTAGTAA	AACCCAAACTCTAAACAAATAATAAT
IGFBP1	2	Chr7: 45927579	45928093	TTGAGTAGGGTTTTGGGTGTATTAGTAA	AACCCAAACTCTAAACAAATAATAAT
IGFBP1	3	Chr7: 45927575	45928093	TTGAGTAGGGTTTTGGGTGTATTAGTAA	AACCCAAACTCTAAACAAATAATAAT
IGFBP2	9	Chr2:217497928	217498172	TGGGGGTTTAGGGTGTTAAG	CTAACCCCTAAAAAACACAAAAAACAT
IGFBP2	10	Chr2:217497846	217498172	TGGGGGTTTAGGGTGTTAAG	CTAACCCCTAAAAAACACAAAAAACAT
IGFBP2	11	Chr2:217497740	217498172	TGGGGGTTTAGGGTGTTAAG	CTAACCCCTAAAAAACACAAAAAACAT
IGFBP3	46	Chr7: −45960433	45960866	AGAAGTAGGGGTGGTTTAGGATA	AAACCCTATATACCAATTTCCC
IGFBP3	47	Chr7: −45960418	45960866	AGAAGTAGGGGTGGTTTAGGATA	AAACCCTATATACCAATTTCCC
IGFBP3	48	Chr7: −45960415	45960866	AGAAGTAGGGGTGGTTTAGGATA	AAACCCTATATACCAATTTCCC
IGFBP3	49	Chr7:-45960400	45960866	AGAAGTAGGGGTGGTTTAGGATA	AAACCCTATATACCAATTTCCC
IGFBP3	50	Chr7: −45960394	45960866	AGAAGTAGGGGTGGTTTAGGATA	AAACCCTATATACCAATTTCCC
IGFBP4	23	Chr17: 38599317	38599681	GGGGTTTAGGTTTAGAGGTATTTTGG	ACCCCCAACCCCTTCCCAAAAAT
IGFBP4	24	Chr17: 38599314	38599681	GGGGTTTAGGTTTAGAGGTATTTTGG	ACCCCCAACCCCTTCCCAAAAAT
IGFBP4	25	Chr17: 38599311	38599681	GGGGTTTAGGTTTAGAGGTATTTTGG	ACCCCCAACCCCTTCCCAAAAAT
IGFBP4	26	Chr17: 38599308	38599681	GGGGTTTAGGTTTAGAGGTATTTTGG	ACCCCCAACCCCTTCCCAAAAAT
IGFBP4	27	Chr17: 38599305	38599681	GGGGTTTAGGTTTAGAGGTATTTTGG	ACCCCCAACCCCTTCCCAAAAAT
IGFBP4	28	Chr17: 38599302	38599681	GGGGTTTAGGTTTAGAGGTATTTTGG	ACCCCCAACCCCTTCCCAAAAAT
IGFBP4	29	Chr17: 38599299	38599681	GGGGTTTAGGTTTAGAGGTATTTTGG	ACCCCCAACCCCTTCCCAAAAAT
IGFBP7	8	Chr4: −57976141	57975928	GGAAAGGGGAGAAATTAGAGGG	TCCTACTCCATCCCCAAT
IGFBP7	9	Chr4: −57976145	57975928	GGAAAGGGGAGAAATTAGAGGG	TCCTACTCCATCCCCAAT
IGFBP7	10	Chr4: −57976152	57975928	GGAAAGGGGAGAAATTAGAGGG	TCCTACTCCATCCCCAAT
IGFBP7	11	Chr4: −57976157	57975928	GGAAAGGGGAGAAATTAGAGGG	TCCTACTCCATCCCCAAT
IGFBP7	12	Chr4: −57976172	57975928	GGAAAGGGGAGAAATTAGAGGG	TCCTACTCCATCCCCAAT

Target CpGs were identified within 500 bp from the transcription start sites of the respective genes

### Bisulphite modification

PureLink Genomic DNA extraction kit (Invitrogen, K1820-02) was used for genomic DNA extraction. EZ DNA Methylation-Gold TM Kit (Zymo Research, Irvine, CA, USA) was used for sodium bisulphite conversion. Approximately 500 ng of genomic DNA was bisulphite modified by incubating at 98 °C for 10 min and 64 °C for 2 h and 30 min. The product was desulphonated, washed and eluted in 10 μl of elution buffer. A 2 μl of bisulphite-modified genomic DNA (approximately 500 ng) was amplified in a PCR mix containing 2 μl of forward and reverse primer, 12.5 μl of FastStart Taq DNA Polymerase (Lifescience Roche, cat no. 12032929001) and 10.5 μl of water. DNA amplification in a thermocycler was performed by following these PCR conditions: 1 cycle at 95 °C for 6 min, followed by 40 cycles of 95 °C for 30 s, annealing temperature of 55 to 59 °C (depending on primer pair) for 30 s and 72 °C for 30 s, followed by 1 cycle at 72 °C for 30s.

### Pyrosequencing

PyroMark Q96 ID (Qiagen) was used for pyrosequencing analysis. A 10 μl of biotinylated DNA obtained with the PyroMark CpG Assays were complexed with 2 μl Streptavidin Sepharose High Performance beads (GE Healthcare) in a solution containing 30 μl of water and 38 μl PyroMark Binding Buffer (Qiagen) per reaction. After vortexing the mixture for 10 min at room temperature, the beads were washed using the PyroMark Q96 Vacuum Workstation (Qiagen). Beads were then washed in ethanol for 5 s and placed for 5 s in 0.2 M NaOH. The beads were further washed for 5 s in PyroMark Wash Buffer (Qiagen) to ensure that only single-stranded DNA remained attached to the beads. Beads were then placed on a sequencing plate containing 12 μl of the appropriate sequencing primers from the PyroMark CpG Assays resuspended in PyroMark Annealing Buffer (Qiagen). The plate was heated for 5 min at 80 °C in a heating block and then allowed to cool for 2 min before loading it on the pyrosequencer. Assay efficiency was validated by 0 and 100 % methylated DNA (CpGenome Universal Methylatated DNA, 10 μg, Millipore, S7821). The methylation data was analysed by Pyro Q-CpG software 1.0.6.

All the reverse primers were biotinylated. The primers used were IGF1F: 5′-GATAGGAAATAGTTGGGGGAATATTTGT-3′, IGF1R: 5′-AATCTACTTTACCCCAATCACTTCAA-3′; IGFBP1F:5′-TTGAGTAGGGTTTTGGGTGTATTAGTAA-3′, IGFBP1R:5′-AACCCAAACTCTAAACAAATAATAAT-3′; IGFBP2F:5′-TGGGGGTTTAGGGTGTTAAG-3′, IGFBP2R:5′-CTAACCCCTAAAAAACACAAAAAACAT-3′; IGFBP3F:5′-AGAAGTAGGGGTGGTTTAGGATA-3′, IGFBP3R:5′-AAACCCTATATACCAATTTCCC-3′; IGFBP4F:5′-GGGGTTTAGGTTTAGAGGTATTTTGG-3′, IGFBP4R:5′-ACCCCCAACCCCTTCCCAAAAAT-3′; IGFBP7F:5′-GGAAAGGGGAGAAATTAGAGGG-3′, IGFBP7R:5′-TCCTACTCCATCCCCAAT-3′.

### Statistical methods

Kolmogorov-Smirnov test was used to determine normality of the data. Data were expressed as median (interquartile range). Comparison between groups for continuous variables was by Student’s *t* test, Mann-Whitney *U* test, one-way analysis of variance and Kruskal-Wallis test for normally and not normally distributed data. Categorical data were compared using *x*^2^test. Univariate analyses were used to investigate the association between birthweight percentiles and different variables. Statistical analysis was performed using Graphpad Prism 5.0 (Sandiego, CA, USA) and results were considered significant if *p* value was <0.05.

## Abbreviations

BMI: body mass index
CpG: cytosine-phosphate-guanine dinucleotide
IGF1: insulin-like growth factor 1
IGF2: insulin-like growth factor 2
IGFBP1-7: insulin-like growth factor binding protein 1 to 7
LGA: large for gestational age
mRNA: messenger ribonucleic acid
SEM: standard error of mean
SGA: small for gestational age
TSS: transcription start site
